# Protein and Water Distribution Across Visual Axis in Mouse Lens: A Confocal Raman MicroSpectroscopic Study for Cold Cataract

**DOI:** 10.3389/fchem.2021.767696

**Published:** 2021-11-15

**Authors:** Yao Li, Yuxing Li, Xi Liu, Yonghong He, Tian Guan

**Affiliations:** ^1^ Tsinghua-Berkeley Shenzhen Institute (TBSI), Shenzhen, China; ^2^ Department of Ophthalmology, Beijing Children’s Hospital, Capital Medical University, National Center for Children’s Health, Beijing, China; ^3^ Department of Life and Health, Tsinghua Shenzhen International Graduate School, Shenzhen, China

**Keywords:** cataract, cold cataract, lens, Raman spectroscopy, protein and water distribution

## Abstract

**Purpose:** The aims of the study were to investigate cellular mechanisms of cold cataract in young lenses of wild-type C57BL/6J (B6WT) mice treated at different temperatures and to test a hypothesis that cold cataract formation is associated with the changes in lens protein and water distribution at different regions across lens fiber cells by Raman spectroscopy (RS).

**Methods:** RS was utilized to scan the mouse lens at different regions with/without cold cataract. Three regions with various opacification along the equatorial axis in the anterior–posterior lens section were scanned. The intensity ratio of Raman bands at 2,935 and 3,390 cm^−1^ (I_p_/I_w_) were used to evaluate lens protein and water distribution. We further determined water molecular changes through Gaussian profiles of water Raman spectra.

**Results:** Three specific regions 1, 2, and 3, located at 790–809, 515–534, and 415–434 μm away from the lens center, of postnatal day 14 B6WT lenses, were subjected to RS analysis. At 37°C, all three regions were transparent. At 25°C, only region 3 became opaque, while at 4°C, both regions 2 and 3 showed opacity. The sum of the difference between I_p_/I_w_ and the value of linear fitting line from scattered-line at each scanning point was considered as fluctuation degree (FD) in each region. Among different temperatures, opaque regions showed relatively higher FD values (0.63 and 0.79 for regions 2 and 3, respectively, at 4°C, and 0.53 for region 3 at 25°C), while transparent regions provided lower FD values (less than 0.27). In addition, the decrease in Gaussian peak II and the rising of Gaussian peak III and IV from water Raman spectra indicated the instability of water molecule structure in the regions with cold cataract.

**Conclusion:** Fluctuation degrees of RS data reveal new mechanistic information about cold cataract formation, which is associated with uneven distribution of lens proteins and water across lens fiber cells. It is possible that RS data partly reveals cold temperature-induced redistribution of lens proteins such as intermediate filaments in inner fiber cells. This lens protein redistribution might be related to unstable structure of water molecules according to Gaussian profiles of water RS.

## Introduction

Cataract is the main cause of blindness among various eye diseases, which has always been a global health issue (Huang and Chen, 2018). Cold cataract is a phenomenon that opacification occurs in young mammalian lenses when cooled, and the whole process is reversible when warmed (Zigman and Lerman, 1964; Zigman and Lerman, 1965; Lo, 1989). By controlling the temperature, cold cataract is a convenient and realizable model for studying cataract and related physicochemical changes in the laboratory (Lo, 1989; Sivak et al., 1992). The formation and components of cold cataract have been studied *via* various methods in the past decades, including laser scanning (Sivak et al., 1992), NMR (Lerman et al., 1982; Lerman et al., 1983), and protein analyses (Broide et al., 1991; Song et al., 2009).

It has been proven that beaded filament is significant to the lens optical transparency (Blankenship et al., 2001; Song et al., 2009). In biochemistry level, α-, β-, and γ-crystallin are related to the formation of cold cataract (Lo, 1989), and aggregation and phase separation of γ-crystallin play the most crucial role (Lerman et al., 1983; Broide et al., 1991). Therefore, γ-crystallin is considered to be the cryoprotein in cold cataract formation (Lerman et al., 1966; Siezen et al., 1985). Both intermediate filaments and crystallins are concerned with the formation of cold cataract. Furthermore, some other researches raised that supermolecular organization accounted for the formation of cold cataract rather than specific lens protein (Loewenstein and Bettelheim, 1979; Lerman et al., 1982). A further study on the mechanisms of cold cataract is still needed.

So far, there are various optical methods involved in interpreting cold cataract based on changes in optical signals. With the laser scanning system, the opacification of the lens can be evaluated for cold cataract by measuring the intensity of scattered light (Benedek et al., 1979; Petta et al., 2008), relative light transmittance (Banh and Sivak, 2004), as well as the equivalent focal length (Sivak et al., 1992). In addition, optical coherence tomography and optical coherence elastography were used to image the cold cataract model, providing structural information and biomechanical properties (Izatt et al., 1994; Zhang et al., 2018).

Raman spectroscopy (RS) is a non-invasive optical technique to determine the existence of certain molecules, which can be used in ophthalmology (Erckens et al., 2001; Lin et al., 2010). In eye lens study, Raman spectra from RS usually provide feature peaks, which are bound to vibrational modes of specific chemical bonds, such as CH_2_/CH_3_ vibration bond (Smeets et al., 1993) and disulfide bond (Ozaki et al., 1987). Furthermore, amino acid contents along the visual and equatorial axes were scanned in pig lens by Raman spectroscopy (Medina-Gutiérrez et al., 2004). For cold cataract study, Raman spectra for different species were acquired to test the changes in protein and water in lens with cold cataract (Ondruska and Hanson, 1983). Changes in the intensity ratio of tyrosine residues were found in the process of temperature alteration (Mizuno et al., 1984). To further analyze water and protein content in different regions of the lens based on acquired Raman spectra, different studies have used RS to scan lens and lens slices at different positions (Bot et al., 1989; Huizinga et al., 1989). However, among previous researches on cold cataract with RS, the scanning region was usually wide instead of focusing on a small range across lens fiber cells.

The lack of organelles is a feature of lens fiber cells. Lenticular water may play an important role in lens opacification. The state of lens water among different species was studied with NMR (Rácz et al., 1979). Free and bound water mass was evaluated in different lens regions with age-dependent and advanced nuclear cataract (Heys et al., 2008), whereas, molecular level of water distribution was rarely analyzed in the formation of cold cataract. RS is qualified to reveal molecular information of solid and liquid H_2_O (Carey and Korenowski, 1998). Raman spectra of water can be analyzed through four of five fitted Gaussian profiles (Crupi et al., 2008; Huang et al., 2009; Baschenko and Marchenko, 2011). These Gaussian peaks are able to provide intra-molecular vibrational distributions of water, and are considered to be various structural types of hydrogen bond in H_2_O (Crupi et al., 2008; Huang et al., 2009).

In order to study the cellular mechanisms of cold cataract formation at molecular level, we used RS to scan young mice lens sections across fiber cells in vibratome section *in vitro* for the first time. In this work, we aimed to test the hypothesis that cold cataract formation is associated with the changes in lens protein and water distribution at different regions. With microscopic imaging, the opacity occurred as temperature decreased from 37°C to 4°C. The opacity size varied under the treatment of different temperatures during the cold cataract formation. Raman spectra for three regions along the equatorial axis of anterior–posterior (A/P) lens section were obtained at different temperatures (4°C, 25°C, and 37°C). Protein and water content distribution was evaluated *via* related Raman vibrational band quantification. The distribution showed unevenness in opacification areas due to cold cataract. To further discover molecular changes in water, all the water spectra were analyzed in terms of Gaussian profiles.

## Materials and Methods

### Animals and Lens Image

For cold cataract occurring in young mammalian lenses, female C57bl/6J wild-type mice (B6WT) at the age of postnatal day 14 were used as experiment subjects. All the disposals during experiments with mice were according to the approval of the Animal Ethics Committee of Tsinghua Shenzhen International Graduate School. Mice were euthanized with a suitable amount of 4% chloral hydrate and sacrificed by injecting an overdose of anesthetic after surgery. Lenses were immediately isolated under a dissecting microscope (Leica MZ 95) and were immersed into phosphate-buffered saline (PBS). Three lenses were treated at temperatures 4°C, 25°C, and 37°C, separately. To observe cold cataract, lenses were imaged with the microscope at different temperatures. Lenses developed full cold cataract from transparency in 4°C PBS solution in about 2 min, and they were reversible to transparent in 37°C PBS solution in about 2 min. Images of lens samples were captured according to the software on the computer related to the microscope. The size of lenses and cold cataract regions were recorded.

### Lens Vibratome Section

According to previous studies, the fixation procedure shows no impact on water and protein content in the lenses (Bot et al., 1989; Huizinga et al., 1989; Siebinga et al., 1992). Before obtaining B6WT lens slices, fresh lenses should be fixed in 4% paraformaldehyde/PBS (PFA/PBS) solution at different temperatures. To examine 4°C cold cataract, fresh lenses were removed from eyeballs in 4°C PBS and fixed overnight in 4% PFA/PBS solution at 4°C. For lens samples treated at 25°C or 37°C, fresh lenses were dissected in PBS at room temperature, and then transferred to 4% PFA/PBS solution at 25°C or 37°C for 3–5 h. After fixation, lenses were washed with PBS three times and embedded in melting agarose gel on a plane block. The procedures of lens embedding and vibratome section are as follows: about 50 μl of melted agarose gel drop was added on the surface of the plane block. A wedge was cut on the edge of the solid agarose gel to hold the lens with the cutting direction of anterior–-posterior (A/P, along the optical axis) plane. Then to glue the lens and agarose gel on the block, about 0.5 μl of superglue drop was added to the wedge *via* pipette without touching the lens. The lens and gel drop were covered with more agarose gel as a whole. After solidification, the block was superglued on the cutting plate of the vibratome microscope (Leica VT 1200S). Then sections with a thickness of 100 μm were cut in a container of PBS. Lens sections around the equator were collected and kept in 4% PFA/PBS solution again for post-fixation for 15 min, then were washed with PBS three times. All the equatorial lens sections were scanned with Raman spectrometer at room temperature.

### Raman Spectroscopic Scanning Across Fiber Cells

A confocal Raman microspectrometer (Horiba LabRAM HR800) was used for Raman spectra acquisition. Under the microscope of ×50, the spectrum grating was 600 lines, and the hole was 100 μm. The excitation laser wavelength was 532 nm with the power of 25 mW, and the spectral resolution was 1 cm^−1^. Through laser focus adjustment, the field of view was approximately 0.8 μm in diameter.

**FIGURE 1:**
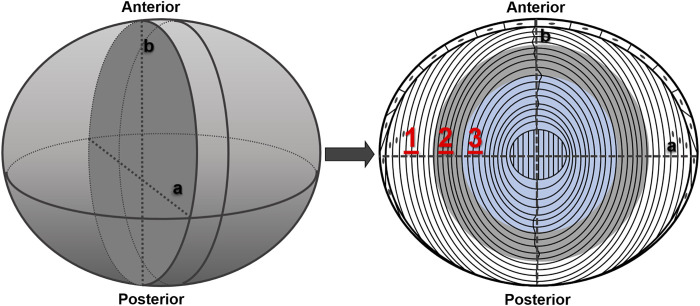
Schematic graph of lens section with different scanning regions. The left plot demonstrates the whole lens after dissection, and the right plot depicts the anterior–posterior (A/P) section of the lens cut as scanning sample. Index lines (a and b) show the orientation of the slice. 1, 2, and 3 in red represents scanning regions 1, 2, and 3 (20 μm in length for each).

Equatorial lens sections were placed on glass slides and covered with cover glasses of 0.14-mm thickness. A drop of PBS solution was added between sliders and cover glasses to prevent lens sections from drying. To scan the lens section across the fiber cells, we set three scanning regions along the equatorial axis vertical to A/P axis (Figure 1) based on the diameter of the lenses and cataract regions under different temperatures. The scanning direction was from cortex to nucleus. For each region, 20 points were collected with the step length of 1 μm, focusing on 50 μm beneath the section surface to avoid uneven interference caused by cutting. In region 1, the lens stays transparent under all temperatures. In region 2, the lens turns opaque at 4°C, while it becomes transparent at 25°C. In region 3, the lens forms cold cataract at both 4°C and 25°C. Raman signal intensity as raw data were acquired for each wavenumber from 2,600 to 3,800 cm^−1^ at each scanning spot. The exposure time was 15 s, with three averaged measurements.

### Statistical Analysis

Statistical significance was evaluated with one-way analysis of variance (ANOVA) with Tukey’s test, using the software Origin (OriginLab, United States). Values pf *p* less than or equal to 0.05, 0.01, 0.001, and 0.0001 were considered statistically significant.

## Results

### Lens Cold Cataract Formation in Microscopic Image

**FIGURE 2:**
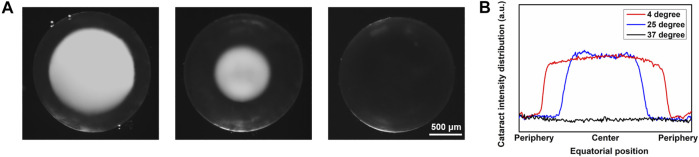
Microscopic image of lens at different temperatures with cold cataract formation. **(A)** Anterior-view images of lens under dissecting microscope. From left to right, lenses were at 4°C, 25°C, and 37°C. Scale bar: 500 μm. **(B)** Plot profile of the equatorial line of the lenses at 4°C, 25°C, and 37°C.

After being immersed in PBS at different temperatures for more than 2 min, lenses were imaged under the dissecting microscope. Three lenses were treated at each different temperature as one control group. As Figure 2A shows, from left to right, lenses were at 4°C, 25°C, and 37°C, separately. At 4°C and 25°C, obvious opacity due to cold cataract formation can be seen. However, the diameter of the opacity sphere at 4°C was much bigger than that at 25°C. At 37°C, no opacity was observed. When the temperature was raised from 4°C/25°C to 37°C, the opacity disappeared in about 2 min, which supported that the cold cataract phenomenon was entirely reversible. There was no significant difference in the formation of cold cataract between the different sexes of mice. Also, there was no noticeable difference between the two lenses from a single mouse.

To quantitatively measure the light scattering distribution due to cold cataract, lens images captured by the dissecting microscope were further analyzed *via* software ImageJ. The plot profile of the gray value in the equatorial section of the lens is illustrated in Figure 2B. The light scattering distribution of the lens apparently differed from each other at 4°C, 25°C, and 37°C, respectively. The sizes of all lenses of the different mice were similar with a diameter of about 1,780 ± 40 μm (n  9). The diameters of the cold cataracts at 4°C were 1,319 ± 28 μm, about 74% of the whole lens diameter. However, the diameter of the cold cataracts, at 25°C was 962 ± 21.0 μm, about 54% of the whole lens diameter.

### Raman Spectra Acquisition and Processing

Based on the different sizes of cold cataract at 4°C, 25°C, and 37°C, regions 1, 2, and 3 were located at about 790–809, 515–534, and 415–434 μm away from the lens center, respectively. To analyze protein distribution at each spot from three regions, three measurements were averaged during signal acquisition. High-wavenumber regions, 2,600–3,800 cm^−1^, were recorded as raw data in this work. Since the Raman intensities of 2,935 and 3,380 cm^−1^ bands represent C–H vibration mode and O–H vibration mode, lens protein and water content can be evaluated *via* these two bands, respectively.

**FIGURE 3:**
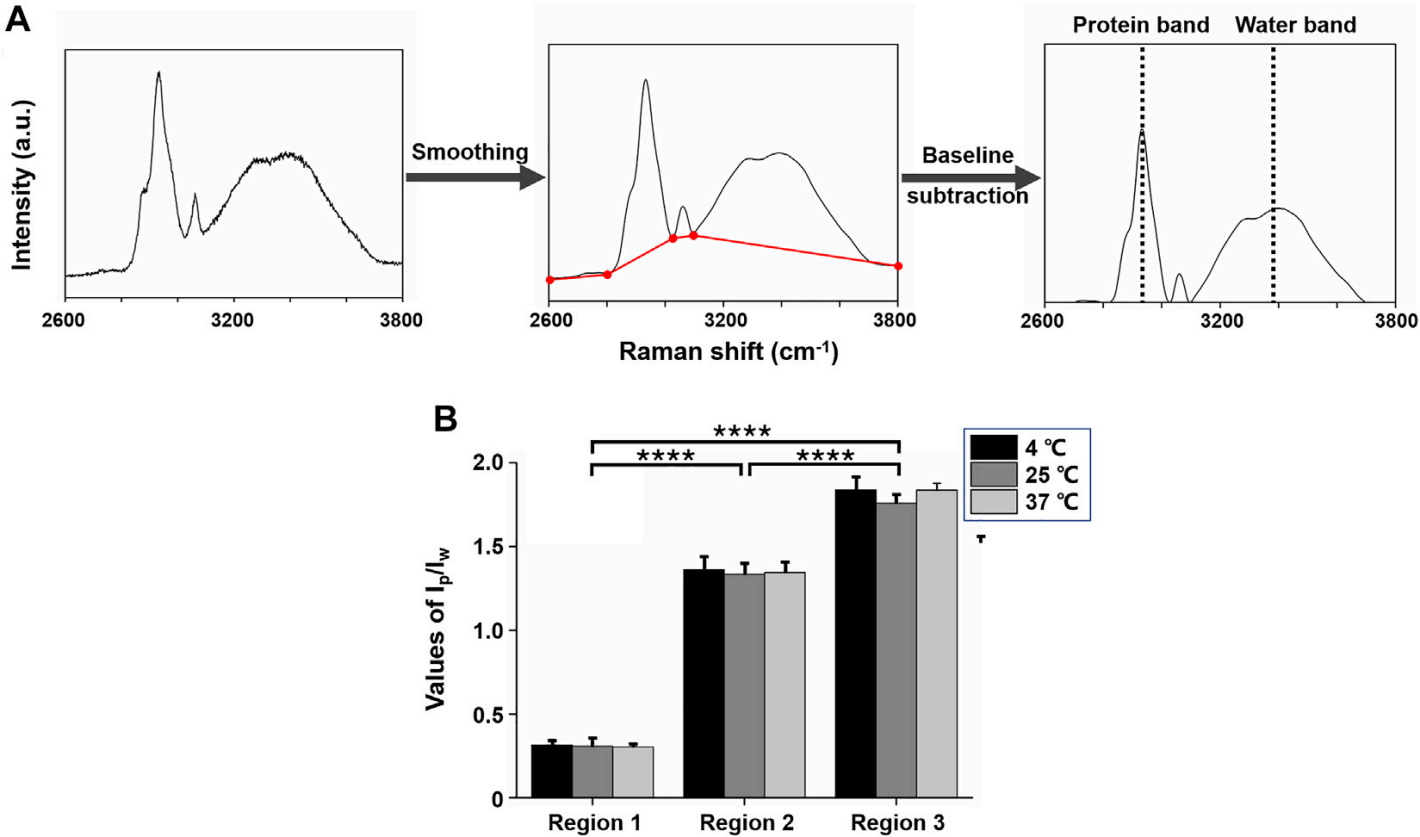
High-wavenumber Raman spectra (2,600–3,800 cm^−1^) of mice lens. **(A)** Spectra processing procedure. **(B)**: Average ratios of the intensity of protein and water brands (I_p_/I_w_) for three regions from all three lenses treated with different temperatures. I_p_/I_w_ values increased from regions 1 to 3 and showed statistically significant differences between each region at 4°C, 25°C, and 37°C (****p ≤ 0.0001).

Raman spectra were smoothed *via* Savitzky–Golay filter. Then background noises caused by fluorescence were subtracted through linear subtraction lines of the intensity of bands 2,600–2,800, 2,800–3,030, 3,030–3,100, and 3,100–3,800 cm^−1^. The procedure of spectra processing is demonstrated in Figure 3A.

### Lens Protein and Water Quantitative Analyses

To quantify protein content distribution in different regions under different temperatures, we evaluated the ratio of the intensity of 2,935 cm^−1^ band as protein content and the intensity of 3,380 cm^−1^ band as water content (I_p_/I_w_) for each scanning point. Figure 3B depicts the average values (±S.D.) of I_p_/I_w_ from all scanning points of the three lenses according to the three regions at 4°C, 25°C, and 37°C. For one lens, in regions 1, 2, and 3, the average I_p_/I_w_ values were about 0.31, 1.35, and 1.81, respectively. This accorded with the rising of protein mass and the decreasing of water mass from lens cortex to nucleus. In regions 1 and 2, I_p_/I_w_ values were not significantly different (*p* > 0.05), which revealed that protein total mass was nearly equal in these two regions with or without cold cataract. However, in region 3, I_p_/I_w_ values were significantly different (*p* ≤ 0.0001).

**FIGURE 4:**
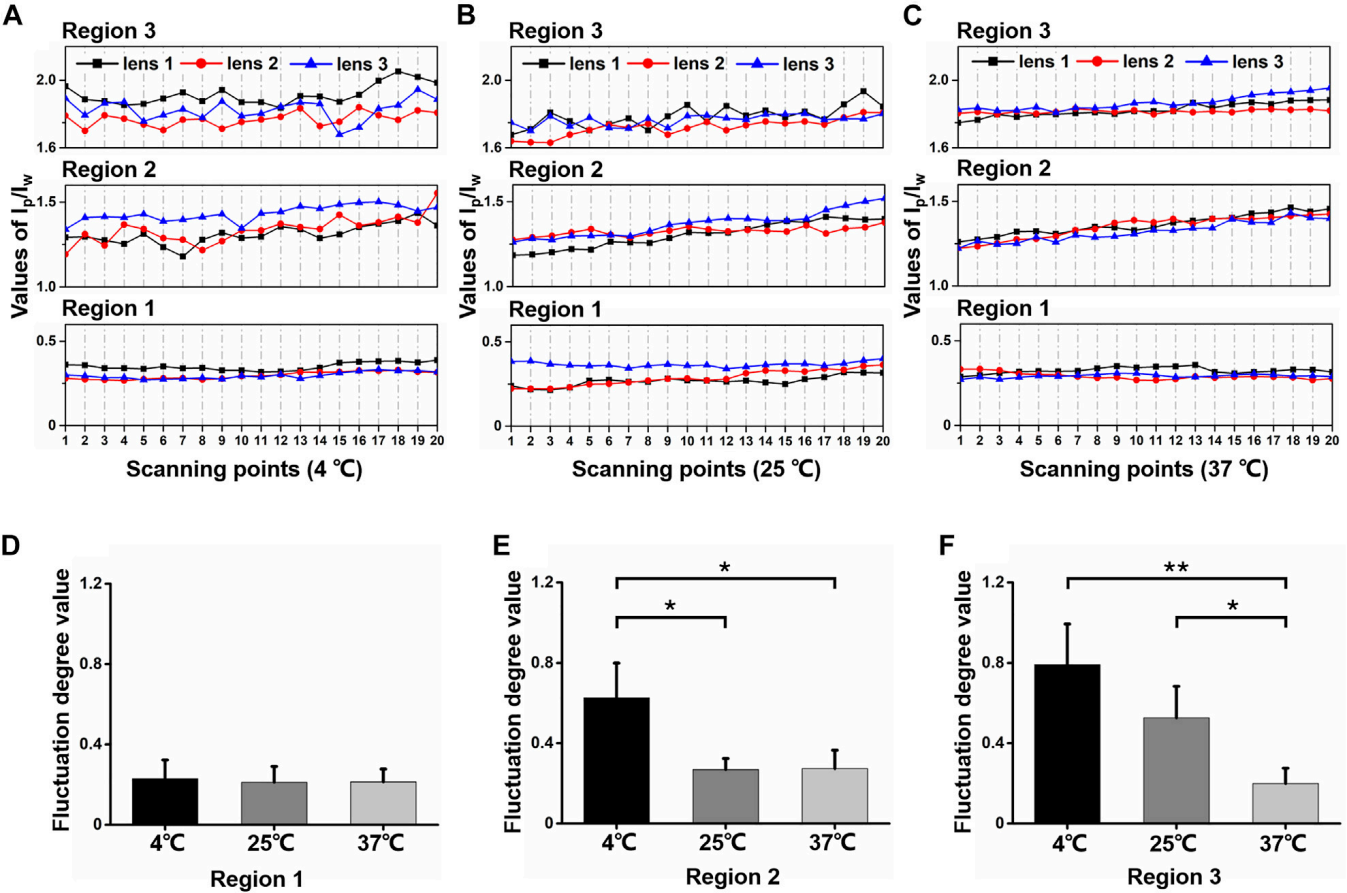
The I_p_/I_w_ values for different regions and different temperatures. **(A–C)** Scattered-line plots of I_p_/I_w_ value for each scanning region at 4°C, 25°C, and 37°C. In each small plot, the display regions of the values were set equal as 0.6. **(D–F)** The value of fluctuation degree for regions 1, 2 and 3 at different temperatures, separately (*p ≤ 0.05, **p ≤ 0.01).

In order to visualize the scanning result from different regions, we demonstrated scatter line plots for I_p_/I_w_ values of every scanning point (scanning points 1–20) in three regions from every lens treated with each temperature in Figures 4A–C. For the 37°C group, scatter lines in all regions were relatively smooth. For the 25°C group, scatter lines in region 3 fluctuated more than those in regions 1 and 2. For the 4°C group, scatter lines were relatively smooth only in region 1, while scattered lines fluctuated obviously in regions 2 and 3. Generally, cold cataract impacted the protein content distribution in different regions under different temperatures.

To further quantify the fluctuation degree (FD), we processed linear fit to every scatter line. The absolute value of the difference between each scatter point and the linear-fit value line were summed up as FD value. As Figure 4D shows, in region 1, FD values were close with an average of 0.22 under different temperatures. However, in region 2, as Figure 4E shows, the FD value was much higher at 4°C (0.63 ± 0.17) compared with25°C (0.27 ± 0.05) and37°C (0.27 ± 0.09). The FD values of the 25°C and 37°C groups were still close. In region 3, as Figure 4F shows, the FD values of the 4°C (0.79 ± 0.20) and 25°C (0.53 ± 0.16) groups were relatively higher than the 37°C group. This revealed that the opacity from cold cataract formation altered protein content distribution among lens fiber cells. The lower the temperature, the more uneven the protein and water content distribution presented with the formation of cold cataract.

### Lens Water Molecular Level Analyses

**FIGURE 5:**
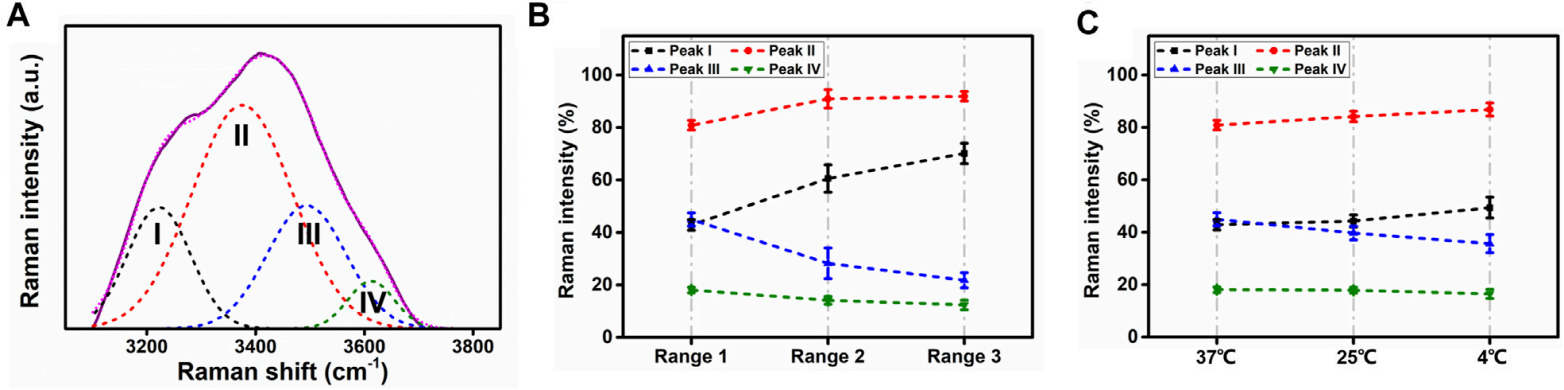
Gaussian peaks and intensity changes without opacity. **(A)** Typical Gaussian fitting components as Gaussian peaks for Raman water spectra, ranging from 3,100 to 3,800 cm^−1^. I, II, III, and IV stand for Gaussian peaks I, II, III, and IV. **(B)** Raman intensity of four Gaussian peaks from three regions at 37°C. **(C)** Raman intensity of four Gaussian peaks at 37°C, 25°C, and 4°C in region 1.

In order to acquire water molecular information from Raman spectra, the water spectra were analyzed through curve fitting with the software Origin (OriginLab, United States). The strategy adopted for the curve fitting procedure was to use well-defined shape components of Gaussian functions, whose peaks were located at about (I) 3,230, (II) 3,400, (III) 3,530, and (IV) 3,650 cm^−1^. All three parameters for each Gaussian function were left to vary upon iteration. The statistical parameters were used as a guide to “best fit” The result of each fitted spectra was characterized by the adjusted R-square of ∼0.99 to ensure the stability of the procedure. Typical Gaussian peaks (peak I to peak IV) are demonstrated in Figure 5A.

At 37°C, the lenses were kept transparent in all regions. The average values of Gaussian peak amplitudes were evaluated according to all the scanning points (three lenses in total, n = 60). Figure 5B depicts the intensity change among the three regions under 37°C. From regions 1 to 3, the intensity of peaks I and II increased, while peaks III and IV decreased.

To reveal temperature dependence in water of normal lens, since there was no opacity in region 1, whether at 4°C, 25°C, or 37°C, Gaussian peak intensities from this region were compared. As Figure 5C shows, from 37°C to 4°C, the intensity of peaks I and II increased as temperature decreased, while peaks III and IV presented the opposite evolution with temperature.

**FIGURE 6:**
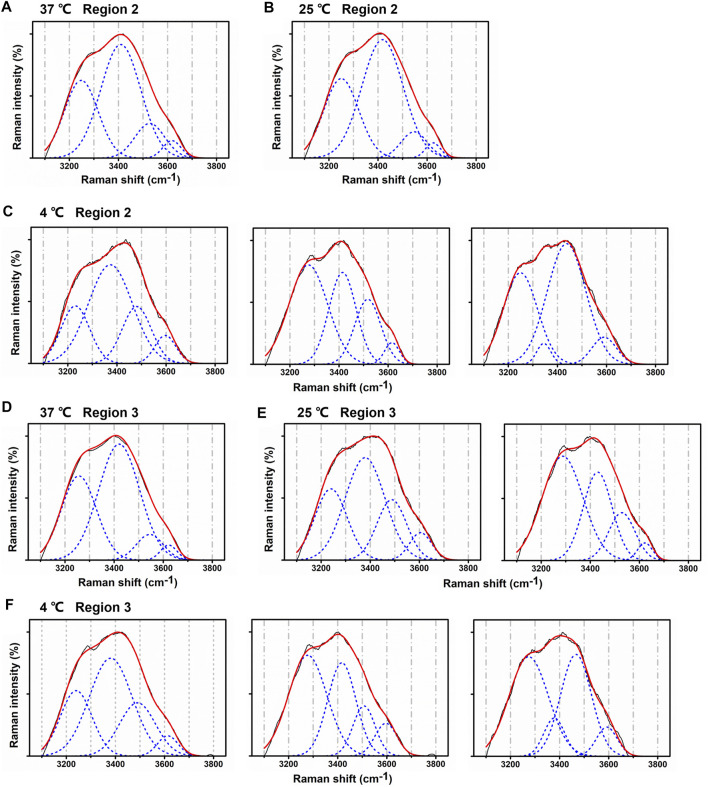
Changes in Gaussian peaks from regions 2 and 3 at different temperatures. **(A–C)** Typical Gaussian peaks from region 2 at 37°C, 25°C, and 4°C. **(D–F)** Typical Gaussian peaks from region 3 at 37°C, 25°C, and 4°C.

Regions 2 and 3 from lenses treated with lower temperature, compared with region 1, presented various patterns of Gaussian components. In region 2, when temperature decreased from 37°C to 25°C, Gaussian peaks were still similar among different scanning points. Figures 6A,B show typical Gaussian well-fitted peaks of the lens water Raman spectra under 37°C and 25°C. However, at 4°C, Gaussian peaks were irregularly compared with 37°C and 25°C. The intensity of peaks III and IV was higher, while peak II decreased at some points. The intensity of peak I was higher or lower at some points (Figure 6C).

In region 3, Gaussian peaks kept fixed relatively only at 37°C, as Figure 6D shows. At 25°C, peaks became fluctuated. The intensity of Peak II decreased, while peaks III and IV increased (Figure 6E). At 4°C, the alteration in peaks was obvious and shared the same regulation with that in region 2 (Figure 6F).

## Discussion

Among previous researches, RS has been utilized to investigate changes in lens with cold cataract. Ondruska et al. studied the formation of dry and cold cataracts from duck, rat, and flounder lenses and found slight changes in Raman spectra (Ondruska and Hanson, 1983). Mizuno et al. investigated the tyrosine doublet in cold cataracts and discovered that some tyrosine residues possessed a change in their hydrogen bonding environment (Mizuno et al., 1984). Other studies basically evaluated protein and water mass in a large range to observe general changing trends (Bot et al., 1989; Huizinga et al., 1989). In this study, we illustrated the various opacity among three regions under different temperatures in P14 B6WT lenses. Region 1 stayed transparent under 4°C, 25°C, and 37°C, while region 2 became opaque only at 4°C. Region 3 became opaque at both 4°C and 25°C. These phenomena and the size of the various opacities with the formation of cold cataract correspond to previous studies from our group (Li et al., 2020). I_p_/I_w_ values represent the protein mass distribution of three regions. Whether at 4°C, 25°C, or 37°C, protein content increases from region 1 to region 3. This result supported that the relative protein to water content of lenses increases from cortex to nucleus (Bot et al., 1989; Huizinga et al., 1989).

Heys et al. analyzed free and total water in human normal and cataractous lenses with thermogravimetric analysis and differential scanning calorimetry (Heys et al., 2008). Through RS, water spectra may not determine the water state in lens, but the structure information of water can be analyzed. In water spectra analyses with Gaussian profiles, peaks I and II represent fully four-hydrogen bonded water molecules, while peaks III and IV are associated to partly hydrogen-bonded free O-H (Crupi et al., 2008). In particular, peak I refers to the in-phase O-H stretching vibrations of hydrogen bonds from adjacent water molecules, while peak II is ascribed to out-of-phase O-H stretching vibrations (Walrafen et al., 1986). In region 1, as temperature decreased, although there was no cataract formation, the intensity of peaks I and II increased, while peaks III and IV decreased. This indicated that O-H in water became more stable with more full hydrogen bonded water molecules, which supported the same change in bulk water and confined water (Crupi et al., 2008). However, when opacification occurred (in region 2 at 4°C, in region 3 at 4°C and 25°C), peaks III and IV increased, while peak II was constrained at some scanning points. The changing patterns of the peaks were irregularly compared with the regions without cataract. These results supported that hydrogen bonds break from water molecules, and out-of-phase O-H stretching vibrations are weakened in lens fiber cells. This corresponded to the evidence that there was lower total water content in the center of advanced nuclear cataractous lenses, and the supported nuclear cataract formation may be associated with lower total hydration of the lens nucleus (Heys et al., 2008).

In this work, we used RS to scan the mice lens in the 20-μm range micron by micron for the first time. By focusing on the protein and water bands of Raman spectra, we discovered that lens opacity due to cold cataract can lead to the fluctuation of protein and water distribution. Compared with regions without cold cataract, in region 3, the I_p_/I_w_ scatter line variance revealed uneven accumulations of proteins at 4°C and 25°C. In region 2, only 4°C of treatment can cause obvious fluctuations in the scatter line. These results showed that low temperature may alter the accumulation or aggregation of filensin proteins in mice lenses, which supported our previous research and the concept of supermolecular organization of protein complexes causing the formation of cold cataract (Ondruska and Hanson, 1983; Li et al., 2020). Furthermore, we used Gaussian peaks of Raman water spectra to investigate cold cataract at the molecular level for the first time. The results supported that hydrogen bonds in water molecules may become unstable and are likely to participate in protein aggregation during cold cataract formation at low temperatures. Mouse cold cataract presents a practical model for understanding the changes in fiber cells during lens development at young ages. Future work is supposed to address the molecular mechanisms on how protein and water distribution become uneven and how water participates in cold cataract formation at low temperatures.

## Conclusion

We scanned across the fiber cells of the lens along visual axis for the first time with RS. By scanning different regions (20 μm for each) with/without cold cataract at different temperatures, protein and water content distribution was quantified. At 4°C, the protein and water distribution of both regions 2 and 3 were uneven. At 25°C, however, only region 3 showed uneven protein and water distribution. At 37°C, all regions were relatively even as comparison. The discovery testified that RS can be utilized to analyze changes in protein and water distribution across lens fiber cells. This proved that cold cataract formation is associated with the uneven protein and water distribution, revealing super-molecular mechanisms. Furthermore, Gaussian profiles of water Raman spectra demonstrated the activation of hydrogen-bonded free O-H in water molecules. The lens protein and water redistribution might be related to the unstable structure of water molecules so that water may participate in this process during cold cataract formation.

## Data Availability

The raw data supporting the conclusions of this article will be made available by the authors, without undue reservation.
